# Microvascular vasodilator properties of the angiotensin II type 2 receptor in a mouse model of type 1 diabetes

**DOI:** 10.1038/srep45625

**Published:** 2017-03-31

**Authors:** Marc-Antoine Begorre, Abdallah Dib, Khalil Habchi, Anne-Laure Guihot, Jennifer Bourreau, Emilie Vessieres, Bertrand Blondeau, Laurent Loufrani, Marie Chabbert, Daniel Henrion, Céline Fassot

**Affiliations:** 1 CNRS UMR 6015, Angers, France; 2 INSERM U1083, Angers, France; 3 MITOVASC Institute, Angers University, Angers, France; 4 CARFI (Cardiovascular Function In Vitro) facility, Angers, France; 5 INSERM UMRS 872, Cordeliers Research Centre, Paris, France; 6 University Hospital (CHU) of Angers, Angers, France

## Abstract

Diabetes Mellitus is associated with severe cardiovascular disorders involving the renin-angiotensin system, mainly through activation of the angiotensin II type 1 receptor (AT1R). Although the type 2 receptor (AT2R) opposes the effects of AT1R, with vasodilator and anti-trophic properties, its role in diabetes is debatable. Thus we investigated AT2R-mediated dilatation in a model of type 1 diabetes induced by streptozotocin in 5-month-old male mice lacking AT2R (AT2R^−/y^). Glucose tolerance was reduced and markers of inflammation and oxidative stress (cyclooxygenase-2, gp91phox p22phox and p67phox) were increased in AT2R^−/y^ mice compared to wild-type (WT) animals. Streptozotocin-induced hyperglycaemia was higher in AT2R^−/y^ than in WT mice. Arterial gp91phox and MnSOD expression levels in addition to blood 8-isoprostane and creatinine were further increased in diabetic AT2R^−/y^ mice compared to diabetic WT mice. AT2R-dependent dilatation in both isolated mesenteric resistance arteries and perfused kidneys was greater in diabetic mice than in non-diabetic animals. Thus, in type 1 diabetes, AT2R may reduce glycaemia and display anti-oxidant and/or anti-inflammatory properties in association with greater vasodilatation in mesenteric arteries and in the renal vasculature, a major target of diabetes. Therefore AT2R might represent a new therapeutic target in diabetes.

Diabetes Mellitus is associated with severe cardiovascular disorders^1^ with a major difference between the two types of diabetes. In type 2 diabetes, usually due to a sedentary lifestyle and excessive food intake, hypertension and atherosclerosis are the major complications faced by patients. In type 1 diabetes, cardiovascular disorders result more from hyperglycaemia with severe nephropathy and strong microvascular alterations^2^.

The renin-angiotensin system has a key role in cardiovascular homeostasis and glucose metabolism. Angiotensin II (Ang II) mainly activates two types of receptors, type 1 (AT1R) and type 2 (AT2R), both coupled to G-proteins. Vascular disorders observed in diabetes are usually attributed to AT1R. Indeed, AT1R blockers or angiotensin converting enzyme inhibition attenuates microvascular complications and nephropathy in diabetic patients^3^,^4^. Altogether, targeting the renin-angiotensin system provides end-organ protection, although this is incomplete^5^. Interestingly, the effects of AT2R usually counteract those of AT1R^6^. Nevertheless, it should be noted that, in ageing^7^ and hypertension^8^,^9^, AT2R has been associated with vasoconstriction in the mesenteric vasculature. However, recent studies have demonstrated a protective role of AT2R in the development of type 1 diabetes^10^,^11^, particularly in the kidney where AT2R stimulation reduced inflammation^11^.

Thus, using mice lacking the gene encoding the receptor AT2R (AT2R^−/y^) and specific pharmacological tools, we investigated the role of AT2R in endothelium-dependent relaxation in the kidney and in mesenteric resistance arteries in a model of type 1 diabetes.

## Results

### Physiological parameters

**Figure 1:**
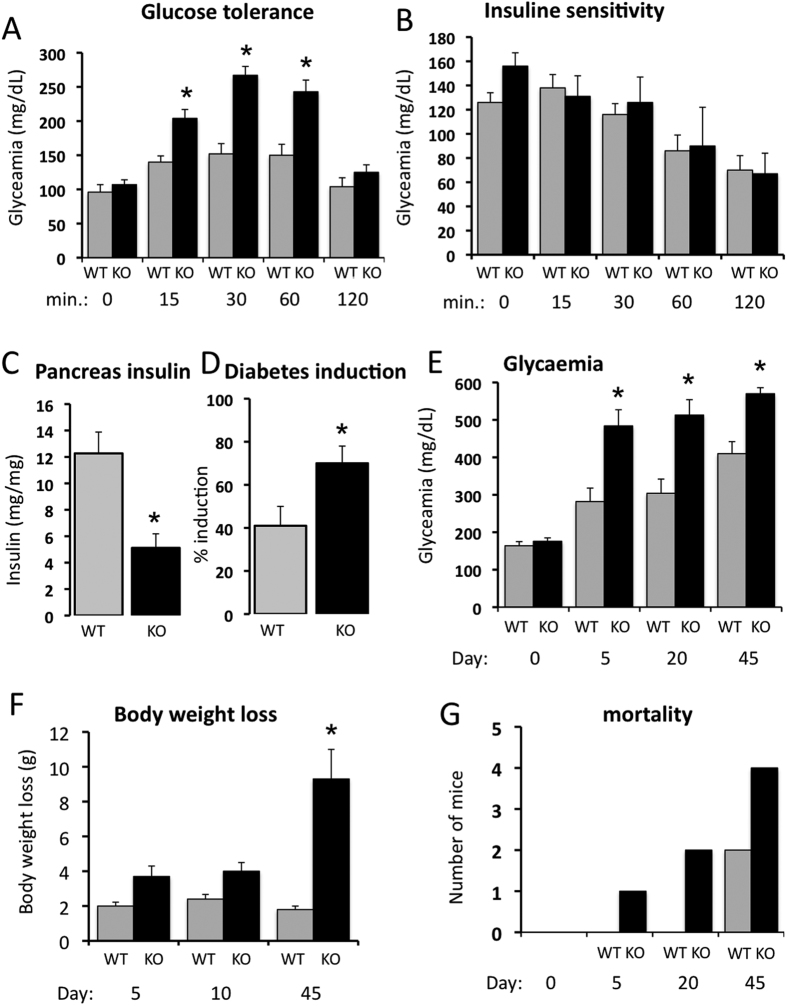
Metabolic profile of the animals. Glucose tolerance (**A**), insulin tolerance (**B**), pancreas insulin content (**C**) and diabetes induction shown as percentage of mice responding to streptozotocin injection (**D**) were determined in wild-type (WT) and AT2R^−/y^ (KO) mice. Glycaemia (**E**), weight loss (**F**) and mortality (**G**) were determined 5, 20 and 45 days after injection of streptozotocin on day zero (0) in WT and KO mice. Mean ± SEM is presented (n = 8 mice per group). *p < 0.05 KO versus WT.

Body weight (31.1 ± 1.3 g vs 28.9 ± 1.4 g) and mean arterial blood pressure (104 ± 4 vs 98 ± 3 mmHg) were similar in AT2^−/y^ and WT mice. Glucose tolerance was reduced in AT2^−/y^ mice compared to WT mice (Fig. 1A), whereas insulin tolerance was not significantly affected (Fig. 1B). Pancreas insulin content was lower in AT2^−/y^ than in WT mice (Fig. 1C). Streptozotocin (STZ) induced diabetes in 40% of WT mice (Fig. 1D) resulted in a progressive increase of glycaemia until 45 days (Fig. 1E). This was associated with moderate body weight loss (Fig. 1F).

Diabetes induction was more efficient in AT2^−/y^ than in WT mice (Fig. 1D) with a higher level of glycaemia 5 to 45 days after injection of STZ (Fig. 1E). STZ-induced diabetes was associated with significantly greater weight loss (Fig. 1F) and mortality (Fig. 1G) in AT2^−/y^ than in WT mice.

### Inflammation and oxidative stress

**Figure 2:**
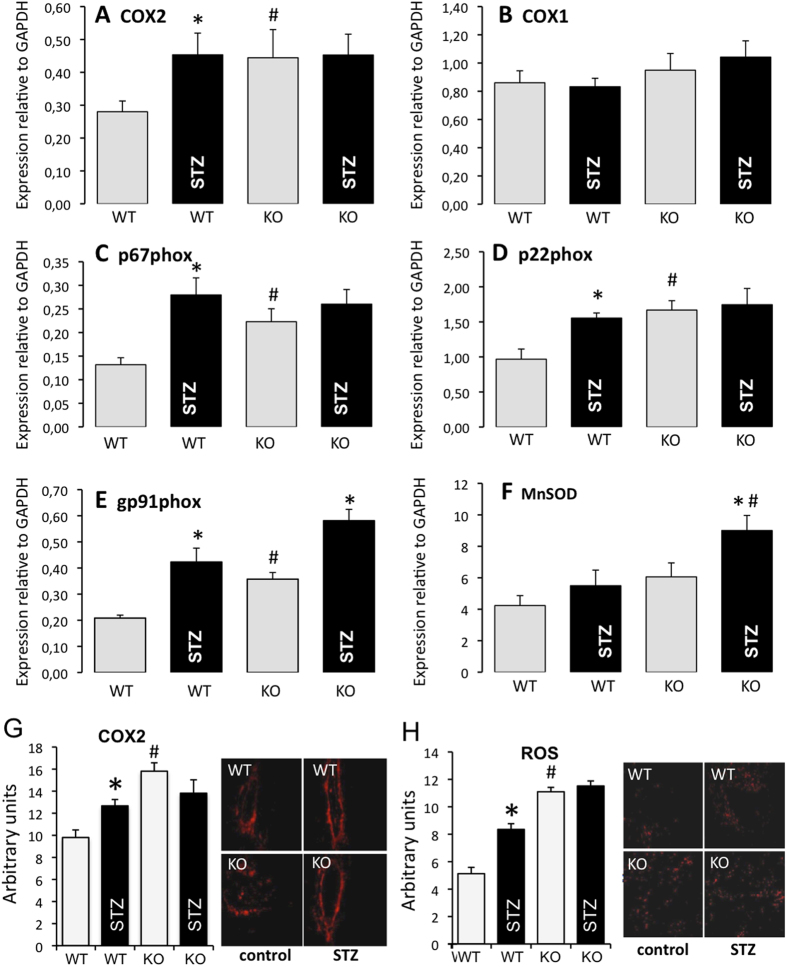
COX and oxidative stress pathways. The mRNA expression level of COX-2 (**A**), COX-1 (**B**), p67phox (**C**), p22phox (**D**), p67phox (**E**) and MnSOD (**F**) was determined in the aorta isolated from wild-type (WT) and AT2R^−/y^ (KO) mice using Q-RT-PCR. Mice were either treated with streptozotocin (STZ, black bars) or not (control, grey bars). Mean ± SEM is presented (n = 8 mice per group). COX-2 expression level (**G**) was determined using immunohistochemistry and reactive oxygen levels (ROS, **H**) using dihydroethidium staining in kidneys isolated from WT and AT2R^−/y^ mice. *p < 0.05, STZ versus control; ^#^p < 0.05, KO versus corresponding WT.

In WT mice, COX-2 gene expression level of aorta (Fig. 2A) was greater in diabetic than in non-diabetic WT mice, whereas COX-1 gene expression level was not affected (Fig. 2B). Similarly, expression levels of p67phox (Fig. 2C), p22phox (Fig. 2D) and gp91phox (Fig. 2E) were greater in diabetic than in non-diabetic WT mice. MnSOD expression level (Fig. 2F) was not affected by diabetes. In the isolated kidneys of WT mice, COX-2 expression, measured by immunohistochemistry (Fig. 2G), and ROS level (Fig. 2H) were higher in diabetic than non-diabetic animals.

Aorta gene expression levels of COX-2 (Fig. 2A), p67phox (Fig. 2C), and gp91phox (Fig. 2E) were greater in non-diabetic AT2R^−/y^ mice than in non-diabetic WT animals. Similarly, in the kidney, COX-2 (Fig. 2G) and ROS levels (Fig. 2H) were greater in non-diabetic AT2R^−/y^ mice than in non-diabetic WT animals.

In AT2R^−/y^ mice, diabetes induction by STZ was only associated with an increased gene expression level of gp91phox (Fig. 2E) and MnSOD (Fig. 2F).

### AT2R and AT1R expression level

**Figure 3:**
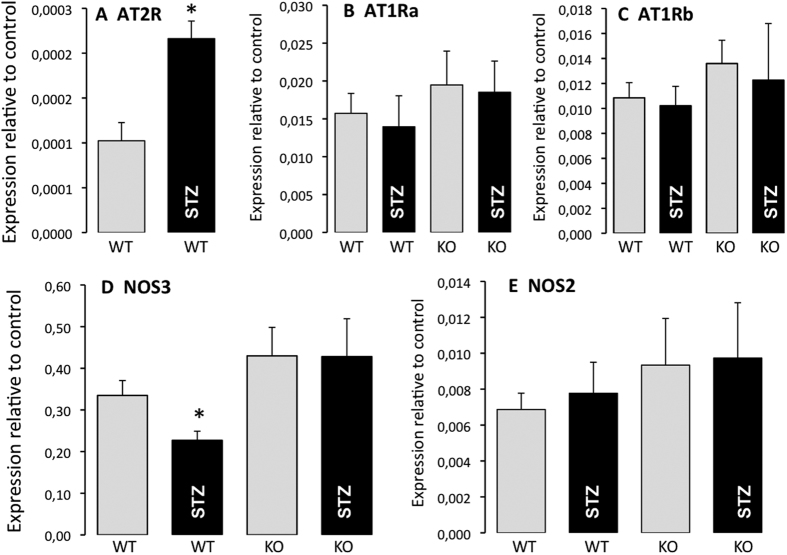
Angiotensin II receptors and eNOS expression levels. AT2R (**A**), AT1aR (**B**), AT1bR (**C**), NOS3 (**D**) and NOS2 (**E**) mRNA expression levels were determined in the aorta isolated from wild-type (WT) and AT2R^−/y^ (KO) mice using Q-RT-PCR. Mice were treated with streptozotocin (STZ, black bars) or were not treated (control, grey bars). Mean ± SEM is presented (n = 8 mice per group). Data is expressed relative to the housekeeping gene (GAPDH, control). *p < 0.05, STZ versus control.

In the aorta, the AT2R gene expression level (Fig. 3A) was greater in diabetic WT mice than in non-diabetic WT animals, whereas the AT1R (AT1a and AT1b) gene expression level was not significantly affected by diabetes in both WT and AT2R^−/y^ mice (Fig. 3B,C).

In the kidney, a similar pattern was observed, although differences were not statistically significant (Supplement S1).

### NOS2 and NOS3 expression level

The gene expression level of NOS3 (Fig. 3D) was lower in diabetic WT mice than in non-diabetic WT animals, whereas it was not affected by diabetes in AT2R^−/y^ mice. Finally, gene expression of NOS2 (Fig. 3E) was not significantly affected by diabetes in both WT and AT2R^−/y^ mice.

### Circulating 8-isoprostane and creatinine

**Figure 4:**
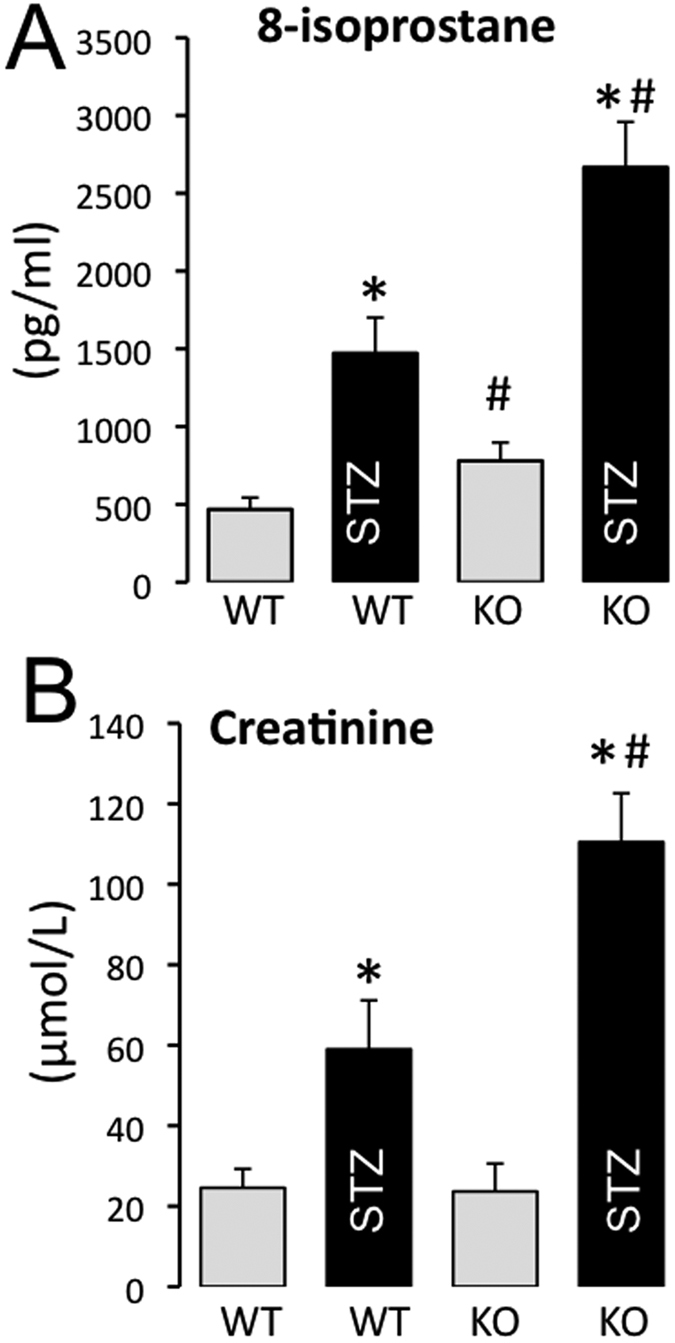
Blood 8-isoprostane and creatinine in mice. 8-isoprostane (**A**) and creatinine (**B**) blood levels were determined in wild-type (WT) and AT2R^−/y^ (KO) mice whether treated or not with streptozotocin (STZ): Mean ± SEM is presented (n = 8 mice per group). *p < 0.05: effect of STZ; ^#^p < 0.05: WT versus KO.

Following STZ-induced diabetes, 8-isoprostane and creatinine levels were higher than in control non-diabetic mice (Fig. 4A,B). Plasma levels of 8-isoprostane were higher in AT2R^−/y^ than in WT mice (Fig. 4A). Plasma levels of creatinine were similar in AT2R^−/y^ and WT mice (Fig. 4B). Moreover, they were greater in diabetic AT2R^−/y^ than in diabetic WT mice.

### AT2R-dependent dilatation in the isolated mesenteric resistance artery

**Figure 5:**
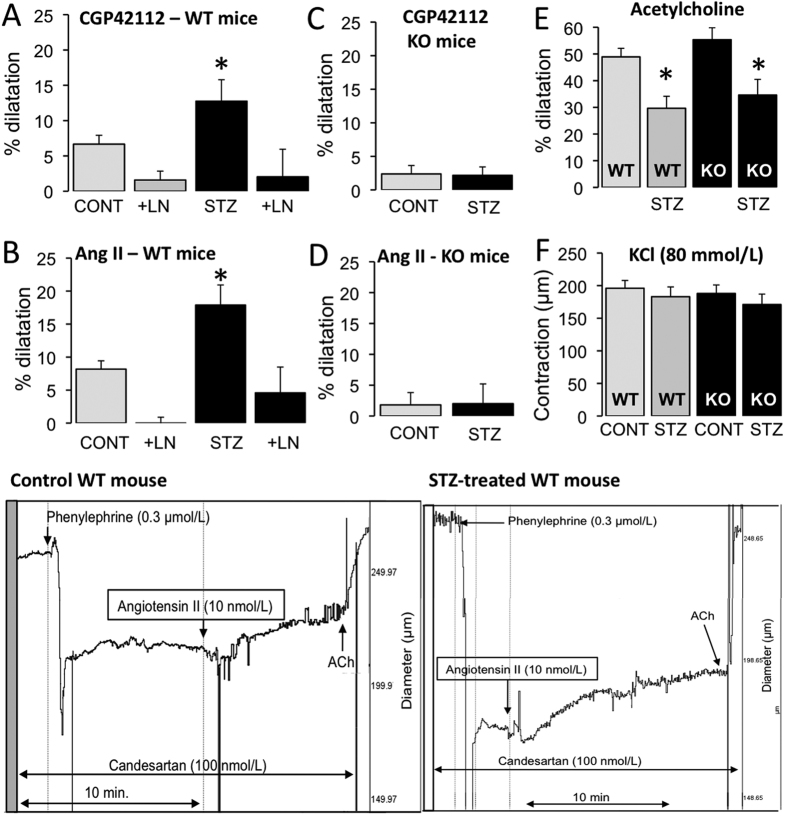
AT2R-dependent dilatation in mesenteric resistance arteries. Vasodilatation was induced by the AT2R agonist CGP42112 (0.1 μmol/L, **A**,**C**) and by angiotensin II (10 nmol/L) in the presence of the AT1R blocker candesartan (100 nmol/L, **B**,**D**) in mesenteric arteries isolated in wild-type (WT) and AT2R^−/y^ (KO) mice whether treated or not (CONT) with streptozotocin (STZ). Vasodilatation was repeated after incubation of the arteries with L-NAME (0.1 mmol/L, LN). Endothelium-dependent (acetylcholine)-mediated dilatation (**E**) and KCl (80 mmol/L)-mediated contraction (**F**) were measured in arteries of WT and KO mice. Mean ± SEM is presented (n = 6 mice per group). *p < 0.05: effect of STZ. Typical recordings show vasodilatation induced by angiotensin II in the presence of candesartan. After stabilizing the response, acetylcholine (ACh) was added to the bath in order to measure maximum vasodilatation. Recordings were obtained from a control WT mouse (left recording) and in diabetic WT mice (right recording).

As AT2R expression level was greater in the arteries of diabetic mice than in those of non-diabetic mice; we measured AT2R-dependent dilatation in mesenteric resistance arteries. Both the AT2R agonist CGP42112 and angiotensin II in the presence of candesartan induced vasodilatation in isolated perfused mesenteric resistance arteries (Fig. 5A,B). AT2R-dependent dilatation was significantly greater in diabetic than in non-diabetic WT mice. In both diabetic and non-diabetic WT mice, the NO synthesis blocker L-NAME abolished AT2R-dependent dilatation. The further addition of indomethacin to L-NAME did not change this pattern (data not shown). CGP42112 and angiotensin II did not induce any significant vasodilatation in arteries isolated from AT2R^−/y^ mice (Fig. 5C,D). Endothelium-dependent dilatation induced by acetylcholine was significantly reduced in both WT and AT2R^−/y^ diabetic mice compared to non-diabetic animals (Fig. 5E). KCl- (Fig. 5F) and phenylephrine-mediated contractions (Supplement S2 and S3) were not significantly affected by the absence of AT2R or by diabetes. Full concentration-response curves for acetylcholine-mediated dilatation and phenylephrine-mediated contraction are shown in Supplementary Figures S2 and S3.

### AT2R-dependent dilatation in the isolated and perfused kidney

**Figure 6:**
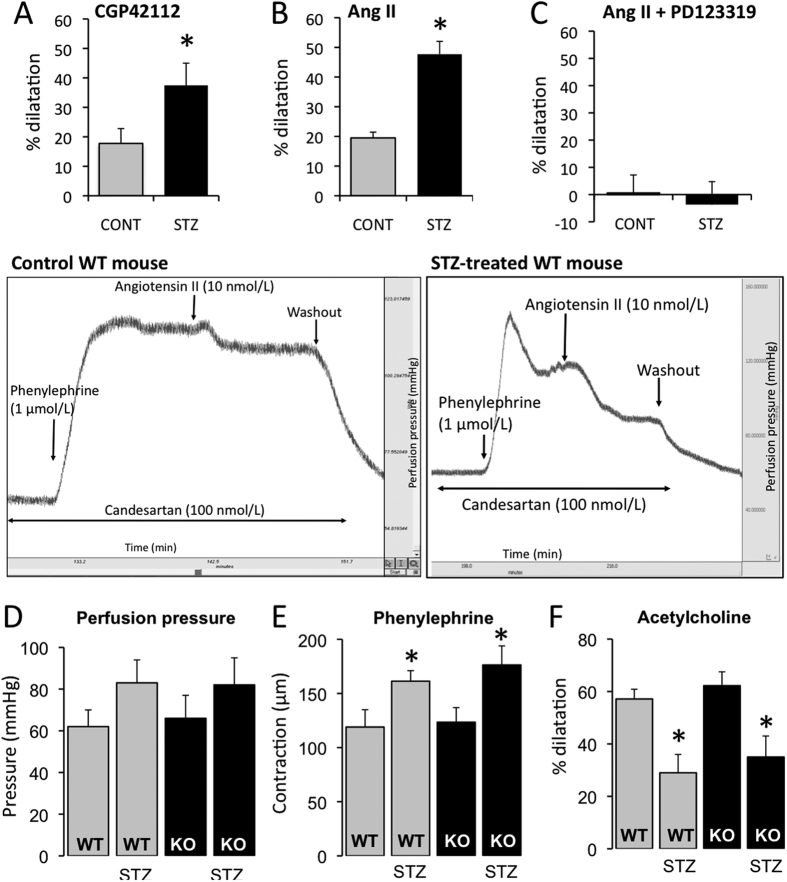
AT2R-dependent dilatation in isolated and perfused mouse kidneys. Vasodilatation induced by the AT2R agonist CGP42112 (0.1 μmol/L, **A**) and by angiotensin II (10 nmol/L) in the presence of the AT1R blocker candesartan (100 nmol/L, **B**) was determined in the perfused kidneys isolated from wild-type (WT) and AT2R^−/y^ mice whether treated or not (CONT) with streptozotocin (STZ). AT2R-mediated vasodilatation was repeated after incubation of the arteries with the AT2R blocker PD123319 (1 μmol/L, **C**). Kidney perfusion pressure (**D**), phenylephrine (10 μmol/L)-mediated contraction (**E**) and endothelium-dependent (acetylcholine)-mediated dilatation (**F**) were measured in WT and KO mice. Mean ± SEM is presented (n = 6 mice per group). *p < 0.05: effect of STZ. Typical recordings show the vasodilatation induced by angiotensin II in the presence of candesartan. Recordings were obtained in a control (non-diabetic) WT mouse (left recording) and in a diabetic WT mouse (right recording).

As the kidney is primarily affected by diabetes, we then investigated AT2R-dependent dilatation in the mouse’s isolated and perfused kidney. We observed that CGP42112 (Fig. 6A) and angiotensin II both induced arterial vasodilatation in the presence of candesartan (Fig. 6B), demonstrated by an increase in perfusion pressure. No effect was observed in AT2R^−/y^ mice (Fig. 6C,D). AT2R-dependent vasodilatation was significantly greater in diabetic mice than in non-diabetic animals. In both diabetic and non-diabetic WT mice, L-NAME abolished AT2R-dependent dilatation and indomethacin had no further effect (data not shown). The AT2R antagonist PD123319 abolished angiotensin II-mediated vasodilatation in the perfused kidney (data not shown). Endothelium-dependent dilatation induced by acetylcholine was significantly reduced in both WT and AT2R^−/y^ diabetic mice in comparison to non-diabetic animals (Fig. 6E); KCl-mediated contraction was not significantly affected in either WT or AT2R^−/y^ diabetic mice compared to non-diabetic animals (Fig. 6F).

## Discussion

Our study suggests a protective role of AT2R in a mouse model of type 1 diabetes. Indeed, AT2R-mediated vasodilatation was greater in the mesenteric resistance arteries and in the kidneys isolated from type 1 diabetic mice.

In type 1 diabetes, hyperglycaemia caused by a lack of insulin leads to cardiovascular disorders resulting mainly from endothelial dysfunction and arterial remodelling as evidenced in human^12^ and in rat resistance arteries^13^. The renin-angiotensin system has a major role in the control of insulin secretion^14^ and in the development of type 1 diabetes^15^,^16^. The role of AT1R in type 1 diabetes has been intensively studied due to its role in oxidative stress and endothelial dysfunction^17^. On the other hand, AT2R stimulation has been shown to protect pancreatic islets through anti-oxidant and anti-apoptotic effects in STZ-induced diabetes in the rat^10^. This observation is further supported by our findings showing a higher level of COX-2, p67phox, p22phox and gp91phox, oxidative stress markers, in AT2R^−/y^ mice regardless of diabetes. This observation reflects previous studies showing that AT2R stimulation prevents endothelial inflammation^18^ and renal oxidative stress^19^. Nevertheless, in diabetic AT2R^−/y^ mice, COX-2, p22phox and p67phox did not increase further in comparison to non-diabetic AT2R^−/y^ mice. In parallel, gp91phox and MnSOD expression levels further increased in diabetic AT2R^−/y^ mice. Thus, the relationship between AT2R and oxidative stress in diabetes requires further explanation. Nonetheless, circulating 8-isoprostane, a marker of systemic oxidative stress increased both in diabetic animals and in AT2R^−/y^ mice. Furthermore, levels of 8-isoprostanefurther increased in diabetic AT2R^−/y^ mice, suggesting that AT2R has a protective role against oxidative stress in the type 1 diabetes model used in the present study. It should be noted that AT2R^−/y^ mice most likely developed compensatory mechanisms, as the knockout is constitutive. Receptors other than AT1R and AT2R such as MAS, AT4R and Mrg or bradykinin receptors are likely to play a different role, yet are better defined in AT2R^−/y^ mice^20^. Nevertheless, our study is in agreement with previous works using a pharmacological approach^11^,^21^,^22^. In these studies, chronic treatment with the AT2R agonist C21 induced a protective effect in STZ-induced diabetes through the inhibition of oxidative stress and reduction of the inflammatory response.

As oxidative stress strongly affects vascular endothelium and kidney microcirculation, we investigated AT2R expression levels and AT2R-dependent dilatation in resistance arteries involved in controlling local blood flow and blood flow in the kidneys, a major target of diabetes. Interestingly, AT2R gene expression was greater in arteries from diabetic WT mice whereas AT1R (A and B) levels were not affected. We tested the direct vasodilator effect of AT2R using angiotensin II in the presence of the AT1R blocker candesartan^23^,^24^ and the AT2R agonist CGP42112^24^,^25^. In agreement with previous reports on animal^7^,^23^,^24^,^25^,^26^ and human arteries^27^,^28^, we observed that the stimulation of AT2R induced vasodilatation in both mesenteric resistance arteries and in the isolated perfused kidney. An important new finding from this study is that AT2R-dependent dilatation was greater in mice suffering from type 1 diabetes. This finding contrasts with previous works performed in rats^29^ and humans^28^ showing that AT2R-dependent dilatation was reduced in type 2 diabetes. One possible explanation could be that the equilibrium between COX-2-derived prostanoids and the NO-pathways is affected differently in type 1 and type 2 diabetes. Indeed, AT2R stimulation in type 2 diabetic rats induced the production of thromboxane A2 which reduced the amplitude of NO-dependent vasodilatation^29^. On the other hand, prostanoids have no significant influence on AT2R-dependent vasodilatation in type 1 diabetes as seen in this study, showing that indomethacin did not affect AT2R-mediated vasodilatation. Indeed, in both the perfused kidney and perfused mesenteric resistance arteries isolated from type 1 diabetic mice, we found that AT2R-dependent vasodilatation involved mainly NO production as L-NAME fully prevented dilatation.

Diabetes is a disease associated with severe vascular disorders, especially in the peripheral circulation such as in the lower limbs and the kidneys. A local increase in blood flow is thus required in these under-perfused tissues in diabetic patients. Most available drugs often block vasoconstrictor pathways in all vascular beds. This is the case for Ang II converting enzyme (ACE) inhibitors, which prevent the formation of Ang II, or sartans, or indeed ARBs, which block AT1R ubiquitously. In both cases, the blockage affects the whole body, and is associated with harmful side effects, related in particular to the inhibition of cardioprotective effects of AT1R stimulation in the heart through a wide variety of β-arrestin-dependent pathways involved in cardiovascular homeostasis^30^. Recently, an arrestin-biased AT1R agonist, TRV027, was shown to be efficient in both reducing hypertension by blocking Gq pathways and improving cardiac contractibility and performance through β-arrestin2-induced pathways^31^,^32^. TRV027 is currently undergoing phase II clinical trials for acute heart failure treatment^33^,^34^. However, its short lifetime (less than 10 minutes) prevents its use for chronic diseases. The search for more stable biased AT1R agonists is an attractive option. However the *in vivo* output of biased ligands is difficult to predict^35^. In view of the findings from this present work, we propose AT2R stimulation in resistance arteries as a therapeutic alternative to inhibiting the renin-angiotensin system. Indeed, AT2R is usually minimally expressed, if not totally absent, in healthy tissues, whereas it may be preferentially expressed in resistance arteries in diseased tissues, as described in the present work and in previous studies^36^. In addition, we have previously shown that AT2R-dependent dilatation does not desensitize and consequently can be repeated over a period of time, in contrast with AT1R-dependent contraction which desensitizes rapidly^25^. This may be related to the absence of β-arrestin recruitment by AT2R and subsequent internalization upon Ang II stimulation, by contrast with AT1R that recruits β-arrestin and internalizes it^37^,^38^. Thus, stimulating AT2R, instead of blocking either the production of AngII or AT1R could be an innovative approach that could be especially attractive in treating diabetes.

## Conclusion

Whereas AT2R has a protective role in the development of type 1 diabetes, AT2R expression level and AT2R-dependent dilatation were also higher in STZ-treated mice. This vasodilator effect was also found in the kidney, an organ that is targeted by diabetes and suffers early and severe damage. Thus, our study highlights a possible protective effect of AT2R in type 1 diabetes.

## Material and Methods

### Animals

Wild-type (WT) and AT2R KO male mice (AT2R^−/y^)^39^ aged 4 to 6 months, were injected with streptozotocin (STZ, 150 mg/kg, i.p., Sigma) in order to induce type 1 diabetes (n = 8 to 16 per group). Body weight and glycaemia (glucometer Accu-Chek Go^®^, Roche) were measured 1, 5, 10, 20, 30, 40 and 45 days after injection. After 45 days, animals were sacrificed by CO_2_ inhalation. The right kidney was quickly cannulated *in situ* and removed from the mouse in order to be perfused as described below. Segments of mesenteric arteries and aorta were then gently dissected and placed in ice-cold physiological salt solution (PSS).

The investigation was conducted in accordance with guidelines from Directive 2010/63/EU of the European Parliament on the protection of animals used for scientific purposes (authorization of the laboratory #00577). The protocol was approved by the Institutional Animal Care and Use Committee (IACUC) and the Committee on the Ethics of Animal Experiments of “Pays de la Loire” (permit #CEEA.2011.14).

### Blood analysis

Plasma 8-isoprostane and creatinine blood levels were determined as previously described using commercially available (Abcam) kits^29^.

### Perfused isolated mesenteric resistance arteries

Arterial segments were cannulated at both ends and mounted in a video monitored perfusion system (Living System, LSI, Burlington, VT) as previously described^40^. Two glass cannulae were used to cannulate a 2–3 mm-long arterial segment. Arterial segments were bathed in a 5 ml organ bath containing PSS with the following composition (in mmol/L): 135.0, NaCl, 15.0, NaHCO3, 4.6 KCl, 1.5, CaCl2, 1.2, MgSO4, 11.0, glucose, 10.0, N-2-hydroxyethylpiperazine-N-2-ethylsulfonic acid (HEPES). The PSS was maintained at pH 7.4, PO_2_ 160 mmHg, PCO_2_ 37 mmHg. Pressure was set at 75 mmHg and arterial diameter was measured and collected continuously using a Biopac data acquisition system (Biopac MP100 and Acqknowledge^®^ software; La Jolla, CA)^41^. Vascular reactivity was tested using KCl (80 mmol/L). Integrity of the endothelium was tested using acetylcholine (Ach, 1 μmol/L) after precontraction with phenylephrine (Phe, 0.3 μmol/L). AT2R-dependent dilatation was then tested as previously described above using CGP42112 (0,1 μmol/L) or angiotensin II (Ang II, 10 nmol/L) in the presence of candesartan (100 nmol/L) after precontraction with Phe (1 μmol/L)^7^,^25^. AT2R-dependent dilatation was repeated after perfusion of the artery with the AT2R blocker PD123319 (1μmol/L), or the NO synthesis blocker N(omega)-nitro-L-arginin methyl ester (L-NAME, 10^−4^ mol/L, 20 min) or L-NAME (10^−4^ mol/L, 20 min) plus indomethacin (10^−5^ mol/L, 20 min).

### Perfused isolated kidney

A separate group of mice was used for this part of the study, as the method requires a rapid isolation of the kidney *in vivo* before perfusion in the appropriate setup. As previously described^42^, the right renal artery was cannulated with a polyethylene catheter (PE-10, 0.28 mm internal diameter, 0.61 mm external diameter, Intramedic, Evry, France). The kidney was then excised and perfused at 37 °C with PSS, without interrupting kidney flow. The perfusion solution was dialyzed and the pH was adjusted to 7.4. Perfusion rate was 600 μl/min and perfusion pressure was measured continuously (PT-F pressure transducer, Living System, Burlington, VT). Vascular reactivity of the renal circulation was tested using KCl (80 mmol/L). Endothelium-mediated dilatation was tested using ACh (1 μmol/L) after precontraction with Phe (1 μmol/L). AT2R-dependent dilatation was then tested as described above using CGP42112 (0.1 μmol/L) or Ang II (10 nmol/L) in the presence of candesartan (100 nmol/L) after precontraction with Phe (1 μmol/L). AT2R-dependent dilatation was repeated following perfusion of the artery with PD123319, L-NAME or L-NAME plus indomethacin.

### Q-RT-PCR analysis

Segments of the aorta or kidneys were dissected and stored in RNAlater^®^ (Sigma) at −20 °C until RNA extraction using the RNeasy^®^ micro kit (Qiagen) was completed. Two hundred ng of total RNA extracted from each artery were used to synthesize cDNA for RT-PCR using the QuantiTect^®^ Reverse Transcription kit (Qiagen) according to the manufacturer’s instructions. The Quantitative real-time PCR reactions were performed on a 7500 Fast Real-Time PCR System (Applied Biosystems) using Power SYBR^®^ Green PCR Master Mix (Applied Biosystems) and gene-specific primers designed using Primer3 online software (Table 1 in the online Supplement). All data were normalized to the Hprt mRNA. Differences in transcript level were determined using the cycle threshold method as described by the manufacturer.

### Statistical analysis

Results were expressed as means ± SEM. Significance of the difference between arteries was determined by one-way ANOVA followed by Bonferroni’s test. Values of p < 0.05 were considered to be significant. Statistical analysis was performed using Graphpad Prism^®^.

## Additional Information

**How to cite this article**: Begorre, M.-A. *et al*. Microvascular vasodilator properties of the angiotensin II type 2 receptor in a mouse model of type 1 diabetes. *Sci. Rep.* **7**, 45625; doi: 10.1038/srep45625 (2017).

**Publisher's note:** Springer Nature remains neutral with regard to jurisdictional claims in published maps and institutional affiliations.

## Supplementary Material

Supplementary Information
