# Supplementary material for: Microvascular vasodilator properties of the angiotensin II type 2 receptor in a mouse model of type 1 diabetes
Source: Sci Rep. 2017 Mar 31;7:45625. doi: 10.1038/srep45625 (PMC5374544; doi:10.1038/srep45625)
Supplement: Supplementary Information [file srep45625-s1.pdf]

Microvascular vasodilator properties of the angiotensin II type 2 receptor in a mouse model of type 1 diabetes.

Marc-Antoine Begorre, Abdallah Dib, Khalil Habchi, Anne-Laure Guihot, Jennifer Bourreau, Emilie Vessieres, Bertrand Blondeau, Laurent Loufrani, Marie Chabbert, Daniel Henrion, Céline Fassot

Online supplement

Supplementary Table 1: references of the genes investigated.

Gene (encoded protein)	NCBI reference Sequence	Forward (5'-3')	Reverse (3'-5')
hprt	NM_013556.2	tgatagatccattcctatgactgtaga	aagacattctttccagttaaagttgag
gapdh	NM_008084.2	ccggggctggcattgctctc	Ggggtgggtggtccagggtt
gusb	NM_010368.1	ctctggtggccttacctgat	Cagttgttgtcaccttcacctc
agtr1a (AT1Ra)	NM_177322.3	actcacagcaaccctccaag	Ctcagacactgttcaaaatgcac
agtr1b (AT1Rb)	NM_175086.3	gtgacatgatcccctgacagt	agtgagtgaactgtctagctaaatgc
agtr2 (AT2R)	NM_007429.4	Taqman probe, Assay ID: Mm01341373_m1 (Life Technologies)	
ptgs1 (COX-1)	NM_008969.3	cctctttccaggagctcaca	tcgatgtcaccgtacagctc
ptgs2 bis (COX-2)	NM_011198.3	gggagtctggaacattgtgaa	gcacattgtaagtaggtggactgt
cyba (p22phox)	NM_007806.3	tgccctccacttcctgtt	gcagatagatcacactggcaat
cybb (p91phox)	NM_007807.4	gaggttggttcggttttgg	gttttgaaagggtgggtgac
ncf2 (p67phx)	NM_010877.4	ccaagacaacttttctggcttc	ttctggggttttgggtctg
sod2 (MnSOD)	NM_013671.3	gacccattgcaaggaacaa	gtagtaagcgtgctcccacac

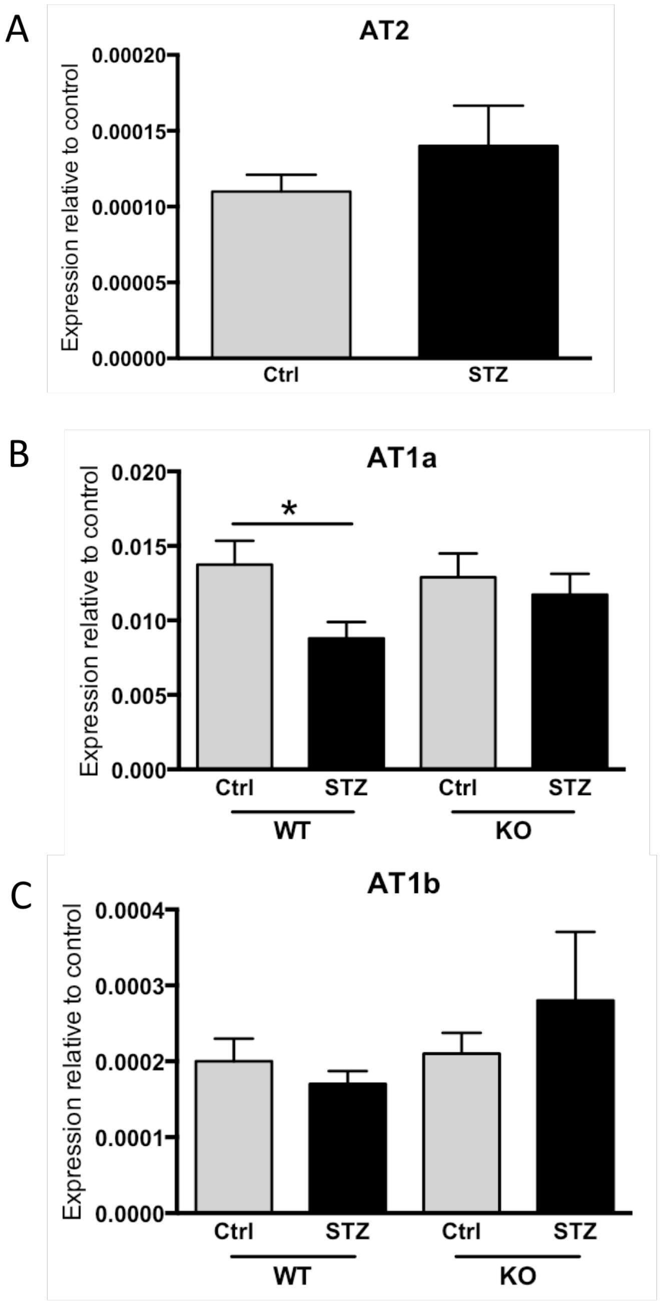

Supplementary figure S1: Angiotensin II receptors expression level in the kidney: AT2R (A), AT1aR (B) and AT1bR (C), NOS3 (D) mRNA expression level was determined in the kidney isolated ofwild-type (WT) and AT2R$^{-/y}$ (KO) mice using Q-RT-PCR. Mice were treated with streptozotocin (STZ, black bars) or not (control, ctrl, grey bars). Data is given as expression rekative to the houvekeeping gene GAPDH (control). Mean ± sem is presented (n=6 mice per group).

* $p<0.05$, STZ versus control.

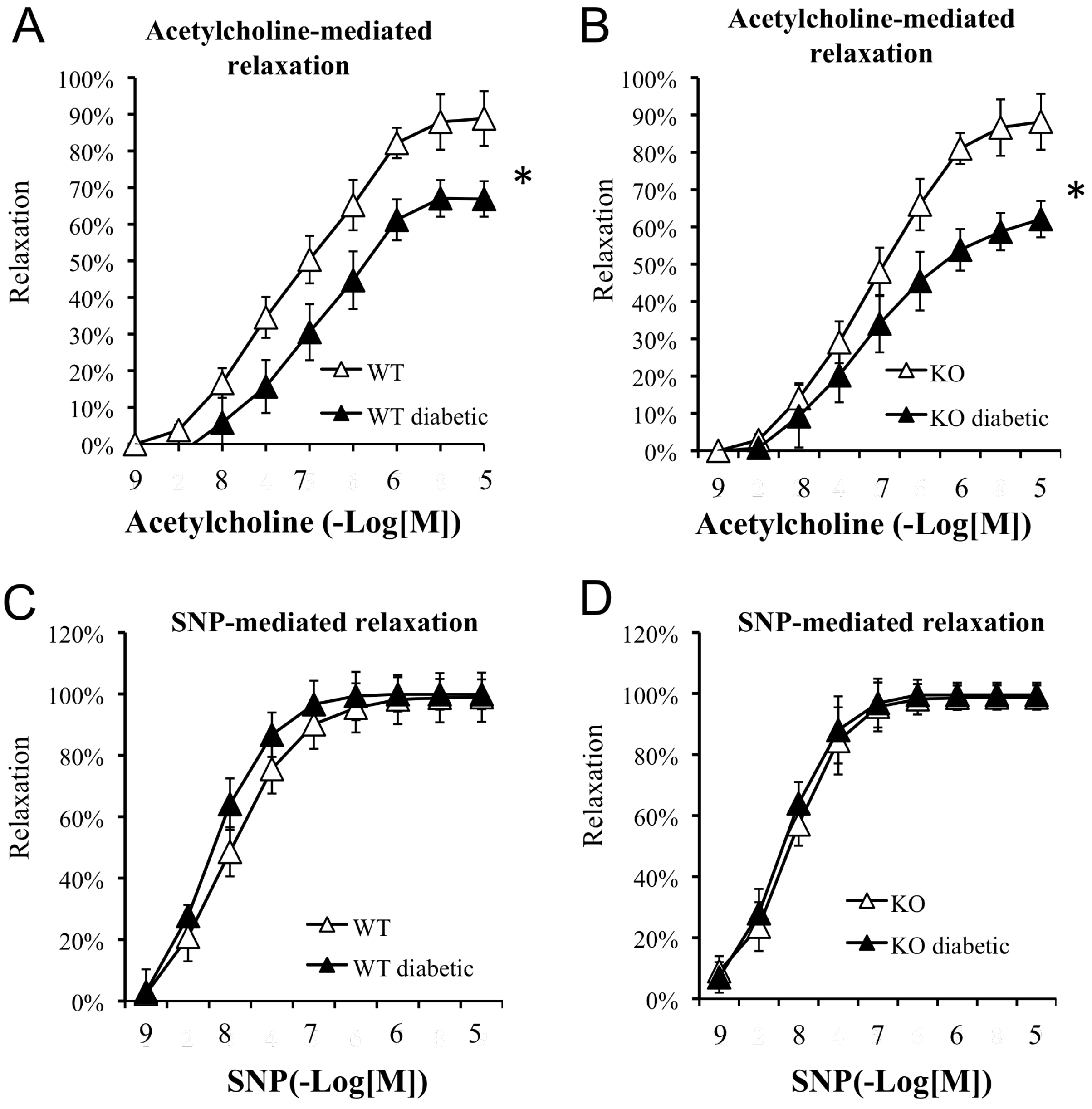

Supplementary figure S2: Acetylcholine (A,B)- and sodium nitroprusside (SNP, C,D)-mediated relaxation measured in mesenteric arteries isolated of wild-type (WT) and $AT2R^{+/-}$ (KO) mice treated with streptozotocin (diabetic) or not.
Mean ± sem is presented (n=6 mice per group)
* $p<0.05$, diabetic versus non-diabetic

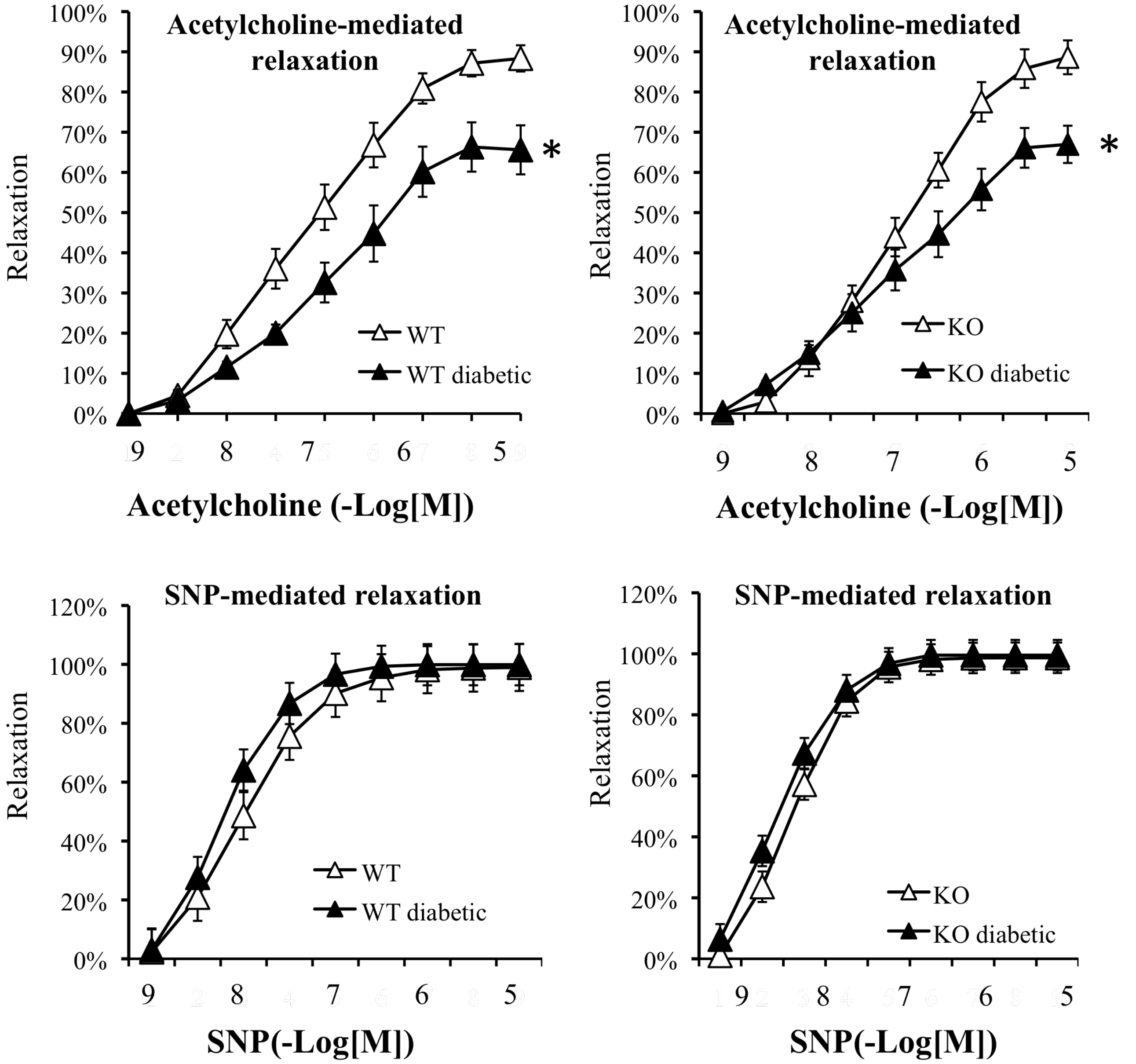

Supplementary figure S3: Acetylcholine (A,B)- and sodium nitroprusside (SNP, C,D)-mediated relaxation measured in aortic rings isolated of wild-type (WT) and AT2R$^{+/-}$ (KO) mice treated with streptozotocin (diabetic) or not.
Mean ± sem is presented (n=6 mice per group)
* $p<0.05$, diabetic versus non-diabetic
